# Prescriptions and Dispensing

**Published:** 1907-11-30

**Authors:** 


### THERAPEUTICS.

### PRESCRIPTIONS AND DISPENSING.

### VI. INSOLUBLE SUBSTANCES IN MIXTURES.

THE second of the imperfect prescriptions is,

℞ Quininæ Hydrochloridi ... ... ... gr. vj.

Sodii Salicylatis ... ... ... ... ʒij.

Ammonii Chloridi ... ... ... ... ʒj.

Tincturæ Gelsemii ... ... ... ... ʒjss.

Aquam ... ... ... ... ad ℥ij.

It is clear that tragacanth should have been included in it; and the method of making up the mixture should be as follows:—Put the tincture of gelsemium into the bottle, and shake so that the inside of the latter is thoroughly wetted with it; then add about 9 grains of powdered tragacanth (remembering that powdered gum tragacanth is not the same as compound tragacanth powder), shake, quickly add 2 ounces of water, and shake again; if preferred, an ounce and a half of tragacanth mucilage might be used instead; dissolve the quinine hydrochloride in half the remainder of the water, add the solution, and shake well; dissolve the sodium salicylate and ammonium chloride in the rest of the water, and add it in two or three portions, shaking well after each. The precipitate of quinine salicylate that is then formed is far more easily diffused evenly by shaking than if no tragacanth were employed; if the quantity of quinine were larger the precipitate would still tend to clot together, and it would be wise to replace the quinine hydrochloride by an equivalent quantity of quinine salicylate, reducing the sodium salicylate by a corresponding amount and adding the equivalent of sodium chloride; the composition of the mixture would be the same as before, but it would now be possible to rub the quinine salicylate to fine powder in a mortar, and suspend it like any other insoluble salt.

If in place of quinine hydrochloride in the above mixture, tincture of quinine were prescribed, the amount of alcohol would then be sufficient to prevent precipitation at once; the medicine would leave the dispensary in good order, but on standing in a cold place crystals of quinine salicylate would be deposited, and the patient might be afraid to take any more of the mixture. This is one of the changes that a doctor who does his own dispensing must foresee; he could obviate the difficulty by using quinine salicylate as just described, and an equivalent quantity of tincture of orange in place of the tincture of quinine. In making up the prescription as it would then stand, the tincture of orange should be added last.

In our third case, namely,

℞ Tincturæ Tolutanæ ... ... ... ʒij.

Vini Ipecacuanhæ ... ... ... ʒij.

Syrupi Scillæ ... ... ... ... ℥ss.

Aquam Cinnamomi ... ... ad ℥iv.

the balsam of tolu becomes thrown out of solution when the tincture is diluted with water, forming an ill-looking mixture with the balsam clinging to the sides of the bottle. It is clear that, if the prescription is to be made presentable, a mucilage is necessary, and, as has been explained above, gum acacia is here indicated instead of gum tragacanth. The prescription would be a much better one if it contained 2 drachms of acacia. The method of dispensing it would then be as follows:—Dissolve 2 drachms of powdered acacia gum in a little of the cinnamon water, and dilute with more up to 2 ounces; stir the tincture of tolu and the syrup of squills together in a measure, and add this in three or four portions to the dilute mucilage, shaking gently after each addition. Dilute the ipecacuanha wine with the rest of the cinnamon water, add, and mix with a final gentle shake.

In these and other examples the young practitioner should not only follow the directions given, but should also vary them and observe the result. Thus, in the bismuth mixture, trials should be made with different quantities of compound tragacanth powder—say, 20, 40, and 60 grains to each drachm of bismuth salt. The effects of other ingredients besides those named should be tried.

#### INSOLUBLE VEGETABLE POWDERS.

It is, of course, by no means the case that all insoluble substances in mixtures require the addition of gum or other suspending agent. Some insoluble salts, like magnesium carbonate, are so easily diffused through the mixture by shaking, and settle again so slowly, that they do not interfere with the proper dose being taken. The same is true of many vegetable drugs of which the powder is prescribed in mixtures—*rhubarb*, for example; in these cases, however, it will not do to put the powder straight into the bottle and shake it up with some of the vehicle. Some powders may contain small lumps which require to be broken down, and all are liable to retain some air, entangled with the particles, which prevents the latter being distributed through the liquid. The mixture will then have a film of dry powder floating on the top, or small bubbles of air coated with powder, and in either case the appearance is bad, and the doses will not be uniform. What we mean can be seen at once on shaking up a little Pulv. Rhei Co. with water in a bottle. It should be a rule without exception to rub down an insoluble powder in a mortar, and it is often best to add to it one of the other ingredients before any of the vehicle. In the following example,

℞ Pulveris Rhei ... ... ... ... ʒij.

Sodii Bicarbonatis... ... ... ... ʒj.

Syrupi Zingiberis ... ... ... ... ʒvj.

Aquam Menthæ Piperitæ... ... ad ℥vj.

the rhubarb and the sodium bicarbonate should be triturated together in a mortar, then the syrup added, and the mixture rubbed up till it is perfectly smooth, a little of the peppermint water being added if necessary; then, still stirring, enough of the peppermint water should be added to make the mixture sufficiently thin to pour easily, when it should be transferred to the bottle, the mortar being afterwards rinsed out with further quantities of the peppermint water.

*(To be continued.)*

